# Staff feedback within an acute mental health hospital: a qualitative interview study

**DOI:** 10.3389/fpsyt.2025.1644734

**Published:** 2025-09-11

**Authors:** Roy Deveau, Jill Bradshaw

**Affiliations:** ^1^ Tizard Centre, University of Kent, Canterbury, United Kingdom; ^2^ Department of Social Policy Sociology and Criminology, University of Birmingham, Birmingham, United Kingdom

**Keywords:** mental health care, staff experiences, feedback, organizational support, multi-professional collaboration

## Abstract

**Introduction:**

Bringing about lasting improvements and change to staff practice in mental health (MH) hospitals is urgently needed but difficult to achieve and maintain. Feedback is a common factor supporting interventions to improve MH practice but does not occur within a *tabula rasa*.

**Method:**

This study explored the existing feedback experiences of 11 staff and managers working in a MH hospital using semi-structured interviews.

**Results:**

Four main themes with sub-themes emerged: Structure and feedback mechanisms varied by role and profession, feedbacks were either formal and procedural or informal during interactive flows, feedback focus and mechanisms differ along professional lines, and feedback can be individualized to roles and personal factors. The overall feedback environment was shown to be complex.

**Discussion:**

Overall organizational feedback interventions may not be effective, and applying the “two systems of thinking” and “teachable moments” ideas to current formal and informal feedbacks may support more effective feedback. Existing formal and informal feedback mechanisms and focus have strengths and weaknesses which can be used to improve care once these have been carefully assessed.

**Conclusions:**

Using concepts such as systems of thinking and teachable moments for linking formal structured and informal “in the moment” feedbacks may present useful approaches to improving professional feedback within MH services.

## Introduction

Deveau (1) notes the difficult and complex roles that staff working in mental health care hospitals undertake. Staff skills, knowledge, and attitude are important in providing quality support to people in mental health hospitals (2). As noted by Kushlick (3), this requires the coming together of disciplines, including direct-care staff, with a shared focus on improving outcomes. Training is one way in which services aim to increase staff skills and knowledge and to improve attitudes (4). However, supporting mental health staff working to implement evidence-based approaches remains challenging (5).

Authors are often not transparent about the theoretical frameworks underpinning their research, but approaches seem to be informed by a number of broad areas. A number of key features seem to be important in bringing about lasting changes that are broadly based within organizational behavior theory. These include combining classroom-based with hands-on training (6, 7), the use of competency-based training programs (which typically include instruction, modeling, rehearsal, and feedback) (8) and organizational support for ongoing staff development (9), such as that provided by practice leadership. Practice leadership (10) is a model of management where managers are focused on developing and maintaining staff support rather than focusing on administrative processes (11). Higher levels of observed practice leadership have been found to be associated with a better quality of support (12).

From an implementation science perspective, previous research has identified senior leaders as making an important contribution to creating a positive working environment, though the picture in practice is rather mixed (13). Leadership interventions may also be helpful in protecting staff wellbeing (14). First-line managers may be ill-equipped to provide practice leadership (15), lacking persistence in approaches, the necessary organization support, ownership, and autonomy. Recent research in health and social care settings (15, 16) has focused on staff feedback as an intervention, an intervention which is broadly drawn from feedback theory. Formal team communication seems to be better than informal *ad hoc* conversations (e.g., those that happen in the hallway) (17). Feedback that was informed by theory (e.g., goal setting theory) was found to be more effective than usual practice (16).

Jones et al. (18) found that feedback from patients could act as triggers to staff reflection, depending on whether the feedback was seen as personally relevant to that staff member and that work was therefore needed to make sure that formal feedback (e.g., data collected by organizationally led surveys) might have limited value in this regard. Sheard et al. (19, 20) found that staff needed to believe that listening to patients was worthwhile and needed to be able to have the resources and organizational support and permission to implement change.

The timing of feedback is also influential (21), with studies suggesting that staff preferred more immediate feedback as they were learning an approach, shifting to more distal feedback after they had acquired the skill. The reactions of the recipient to feedback have been found to influence both the accuracy and the amount of feedback given (22), with positive reactions maintaining the accuracy of feedback and neutral or negative reactions decreasing the accuracy and frequency of feedback.

Feedback is an important component of many interventions to improve MH care, e.g., audit and compliance with support plans. Providing feedback is not undertaken within a *tabula rasa*, e.g., existing staff experiences, differing professional work practices, and cultures will influence responses to feedback. Therefore, the aim of this research was to understand frontline staff experiences of feedback within an acute MH hospital. The results may inform managers and practitioners what feedback methods are likely to be effective with frontline staff. The results may also suggest to researchers useful areas for further investigation.

## Methods

Semi-structured interviews were conducted with 11 frontline staff; these comprised a sample of convenience. Interviews were audio-recorded, transcribed, and analyzed using thematic analysis. A pragmatic research framework (23) was used to conduct the study.

### Participants and setting

The research setting was a MH hospital operated by an independent health and social care provider based in Wales. The hospital provided for a maximum of 40 patients within four separate units, one of which was locked. Healthcare was provided by a total team of around 150 staff supported by clinical psychologists, psychiatrists, and occupational and speech therapists.

The staffing for each shift comprised two registered mental health nurses (staff nurses) with one acting as the “medication nurse” and the other one as shift leader. The shift leader was also responsible for managing/coordinating clinical decision processes, e.g., multidisciplinary meetings (MDT) and reviews. The patient’s care and progress was monitored and planned through daily MDT meetings. Each unit had one or two senior support workers (SSW—with no nationally recognized training) who allocated and monitored a daily support worker (SW—with no nationally recognized training) with tasks and patient activities and routines over the 12-h shift. The number of support workers per unit varied. Some patients were on enhanced observation, with one and sometimes two SW undertaking continuous observation. Daily psychology support for patients was provided by assistant psychologists (AP) and positive practice workers (PPW), both supervised by a clinical psychologist. AP have a frequent presence on units, and PPW have a dual role of providing psychology input and as SW.

### Procedure

#### Ethical approval

As service evaluation, ethical procedures were followed as agreed by the organization’s ethics review process. This included gaining informed consent, anonymity for participants, and data collected in privacy using audio-recorded semi-structured interviews.

#### Data collection and analysis

Interviews and analysis were conducted by the primary researcher and first author who had no prior association with the hospital. Interviews were conducted over 4 days in May 2024 (see **Table 1**) using a conversational style. Material describing the purpose and expectations upon participants, in addition to a consent form, was sent to the hospital and distributed to the staff by the managers. This material included contact details for staff interested in participating to contact the primary researcher (first author) for further information and complete the consent procedures prior to the interview dates. No potential participants replied despite a follow-up circulation of material, although (anecdotally) some staff expressed an interest in being interviewed. A senior manager contacted wards at the time of data collection to enable staff who had expressed to participate and seek volunteers from staff who were “on shift”, had read, or had heard about the material. They were able to be released from shift and were happy to discuss participation in person with the researcher. Inclusion was based upon having been informed of the research as well as being willing and available for interview during the data collection period.

**Table 1:** Staff interviewed and unit staff summary.

Role	Number per shift	Summary of role	Number interviewed
Support workers (SW)	A total of 80 provided day and night care for the whole hospital	Provide patients daily support and observations	None
Senior support workers (SSW)	1 or 2 per unit	“Run the shift” organizing SW tasks and patient activities	One
Positive practice workers (PPW)	1 per unit, on shift or “clinic day”	Split role, three shifts per week as SW which includes “supernumery” hours within each shift for clinical work; 1 day per week “clinical” AP role	Two
Assistant psychologist (AP)	1 for each unit	Clinical work with patients or report writing, largely working on a unit	Two
OTs and speech therapists	Cover the whole hospital	Available for consultation	None
Mental health nurses (staff nurses)	Two on shift per unit	One as “medication nurse”; one as “shift leader”	Three: One senior staff nurse, one newly qualified, and one in training (first year)
Teacher	1 covers the whole hospital	Provides educational activity for patients	One
Senior decision makers	NA	Several hospital-based, e.g. unit managers, clinical and operational directors providing professional support and oversight	Two: One hospital-based and one offsite

An interview topic sheet was devised by the first author which guided the initial interview questions. The first interview demonstrated that this prepared topic sheet was not useful due to the very limited relevant experience of the participants. A pragmatic approach was taken, and the subsequent interview questions sought to explore the general feedback, e.g., formats, and focus experienced by frontline staff. The questions always started with a general question, e.g., “Can you tell me about who gives you feedback on your work and what it is about”? The follow-up questions differed depending upon a participant’s response. With subsequent questions seeking further details focused upon what feedback covered, e.g., “What sort of things are discussed with your manager and when”?; who the participant may provide feedback to, e.g., “When you are organizing and managing a shift, what sort of feedback do you give to support workers?”. These questions covered all people that would provide feedback to or receive feedback from the participant in categories of manager/clinicians, peers, and patients. It also included settings and formats where this feedback occurred, e.g., “Do you get any of your feedback in writing or is it all verbal ‘on the spot’?” Interesting lines of enquiry arose throughout the interviews which led to additional questions—for example, to understand how feedback was affected by the structure of frontline staffing. The interviews lasted around 30 min, with the longest being 47 min and shortest 18 min.

Audio data was transcribed and then entered into software package NVivo 12 and analyzed for themes following a reflexive process described by Braun and Clarke (24, 25). Reflexivity and being grounded in the data was supported by both researchers’ lack of recent experience in MH hospital settings. The primary researcher read the transcripts several times, recording notes and ideas for what seemed important to participants. These notes were amalgamated into a preliminary thematic structure. A second researcher (second author) read the transcripts “blind” to the first author’s notes and preliminary themes, also noting potential themes. The two researchers then shared and discussed their notes and preliminary themes. This led to several modifications of views taking time and effort to remain grounded in the participant’s transcripts. The final thematic diagram and structure was agreed by both authors.

A report of the first author’s thematic analysis was sent to all participants with a form asking the participants if they felt that the results represented their views. One participant responded with an email saying it was a “beautiful and accurate report”. A second copy with a reminder was sent with no further responses.

## Results

**Figure 1:**
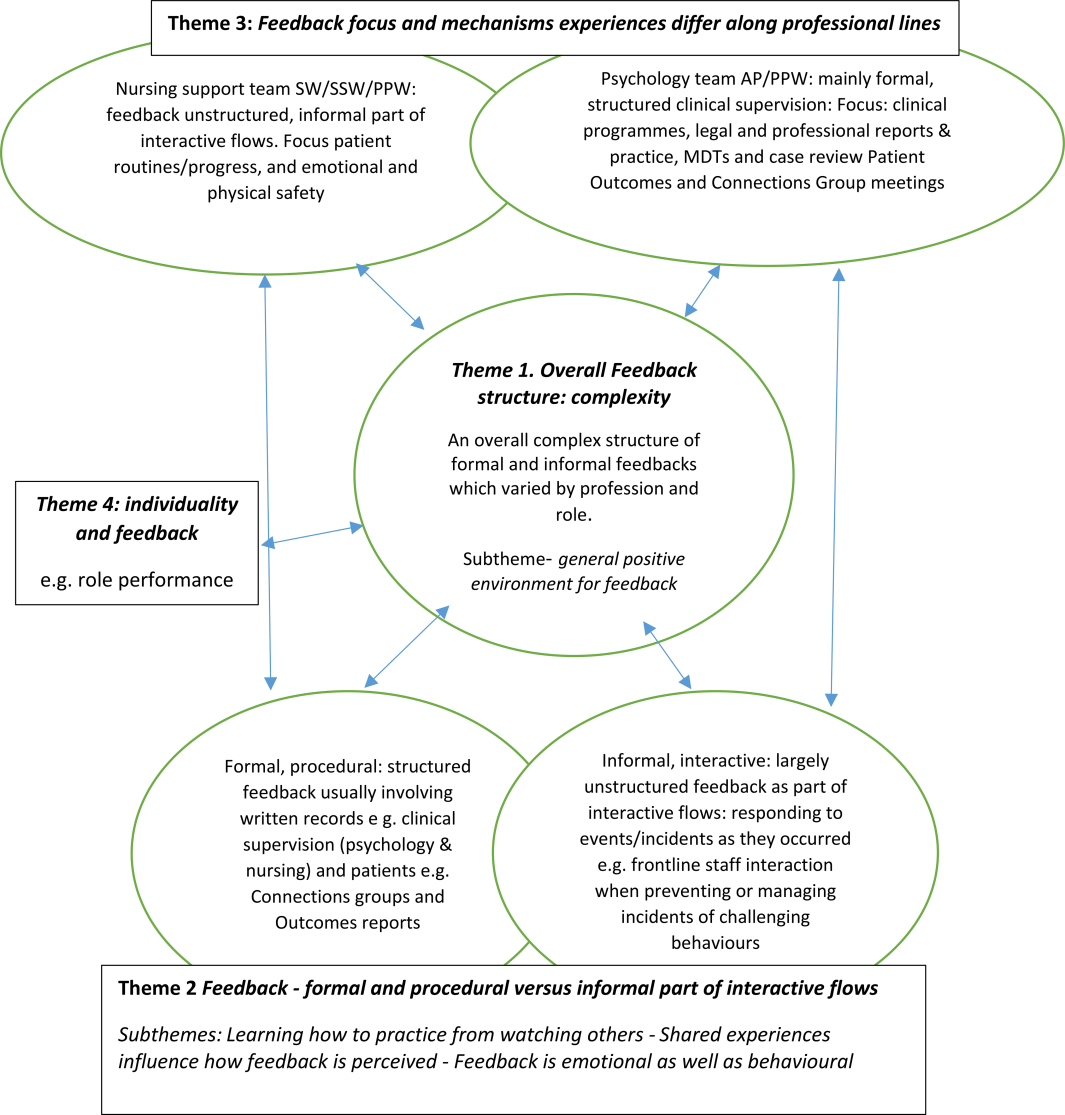
Overall thematic framework.

The themes below (see **Figure 1** for overall thematic framework) are supported by textual evidence from participant interviews which are presented as indented and bold text, denoted by P and a number, e.g., P4, and the interviewer text is signified with I:.

### Theme 1: overall feedback structure: Complexity

The participants described a *complex* range of regular processes and procedures through which feedback was experienced—for example, routine scheduled individual supervision meetings, staff team meetings, group and individual meetings of patients and staff, e.g., connections and outcomes, scheduled therapeutic sessions, e.g., clinical work, and regular surveys of staff and patient experiences and views. Two broad contexts and associated forms of feedback emerged from the data: feedback during immediate *interactive flows* within frontline nursing support teams (e.g., SSW) and more formal feedback within the psychology team (e.g., PPW).

Feedback was received and given through various mediums: verbal as part of everyday interactive flow or verbal and written as part of a structured planned interaction with written records, e.g., regular formal monthly supervisory sessions. Written-only feedback was provided usually as clinical psychologist reviews of important records/reports, e.g., HR20s and clinical program records. A variety of regular meetings were reported by participants as important sources of feedback: the most important one for frontline staff and patients being the weekly unit-based Connections Groups, providing direct feedback on relationships and activities involving staff and patients. A daily unit meeting between various professionals and unit-based staff (referred to as a huddle) was used to feedback patient progress and make decisions regarding their daily care and treatment:

**P2: In our huddle we have 2 nurses, the RC, somebody from OT, somebody from psychology (…) So every morning we have it, they discuss the events from - say today’s huddle would be about the events from the weekend (…) so we would have had a full run down of any incidents of anything that happened over the weekend. All our plans are for today, so we have a patient on a reduction plan and yesterday they would have started general observations, so usually that’s something we would discuss in the huddle,”**

A variety of other meetings based at various levels suggested a complicated structure of meetings to accommodate a variety of staff roles and feedback needs:

**P2: Yeah, we have senior support worker meetings, I know the nurses have nurse meetings, then we have team meetings where everyone’s involved, so there’s always an opportunity to speak up if you’re not feeling comfortable.”**

A quarterly patient outcomes survey reports provided feedback on what patients thought about their care and treatment, e.g., of their structured therapeutic (e.g., psychological) interventions. Two participants mentioned organization-level formal feedback such as awards, e.g., “recommend a colleague”. Patients were often referred to as important sources of feedback, both informally as part of interactive flows (especially for frontline support staff) and more formally through Connections Group and patient outcomes reports:

**P6: Yes, the feedback is documented. The book for the Connections groups. (…) There’s a book we document the meetings and whenever they raise something we bring it forward to the unit manager (….) if there’s feedback say for the night staff doing something we bring it to the attention of the team.”**

Patients were also sources of feedback through their “observed” engagement with (clinical) program of support. This could be feedback to AP/PPW via support staff.

**P5: I guess its indirect feedback where patients are actually using the skills and things that you told them to use, so you can see that something you’re doing is working. Other times, patients will say “my perspective has changed on what’s happened to me and I’ve got hope for the future.”(……) it’s a quarterly thing (outcomes report) to sum up how things are going for the patients and one of them is directed specifically at therapeutic experiences.**

At the extreme of distant feedback (usually experienced by senior decision-makers/clinicians), information was gained from monitoring (online) communications regarding a specific intervention to judge effectiveness, e.g.:

**P11: I will be asked to be kind of copied into the email communication mainly, so that as the case progresses I’ll give people advice and monitor the discussions that are going on around it and then I’ll find out if my advice is accurate and followed. Or sometimes that it hasn’t been accurate, and we’ve needed a different kind of approach. (….) I have to do lots of communication via email (….) But then other times I’ll get feedback when I’m going around, I’ll be asking about a case that I’ve contributed to and then I’ll often get feedback from the managers and senior clinicians because they’re (….) complex cases.**

#### Subtheme: general positive environment for feedback

The hospital setting and overall organization have sought to build an atmosphere that encourages positive and open feedback to enhance patient safety. A range of organization interventions had been implemented, e.g., “see it say it send it” and “recommend a colleague”. Most participants did not refer to these, and those that did not feel that these had influence. However, their overall impact was a shared experience of generally positive working environment, e.g.:

**P6: Yes, sure, they do it in my supervision. It sounds too positive, but my experience has been very positive.**

**P2: I’ve been here now, like I said, a long time, so I’ve always said to the staff “look, I know myself, I know how I can be (stern and off putting) at times. I don’t mean to offend anybody and if I do, come and tell me and I will apologize. Also I expect you to tell me out as well, as I don’t want you to thinking because I’m a senior, you can’t tell me I’m never wrong. I need you to tell me that. If I’m not being told, I’m going to continue to do it wrong.**

In summary, feedback in this setting was communication via a range of mediums and with varied proximity to immediate events. This ranged from being immersed in managing an “incident” to attending an MDT meeting or reviewing emails. Frontline staff were in the main part of immediate interactive flows, and senior decision makers were more distant (see **Table 2**).

**Table 2:** Proximity to daily patient and frontline staff interaction and feedback mechanisms.

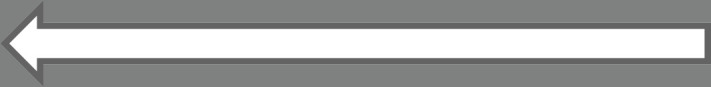 Senior manager & clinicians			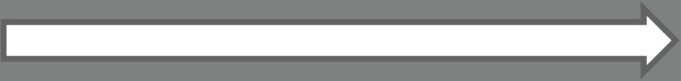 Support & seinor support workers, PPW		
Very distant		Less distant			In the interactive flow
Monitoring email threads. Situations largely analyzed objectively	Reviewing and commenting on reports	Attending next-day de-brief meetings without involvement in the incident	Attend and support de-brief meeting soon after incidents on units	Immediate emotional support, e.g., asking the staff present “are you OK? Do you need 5 min out of it?”	Managing/responding to incidents. Keeping routine activities running smoothly. Fast moving situations often responded to intuitively

### Theme 2: feedback—Formal and procedural versus informal and responsive part of interactive flows

A majority of participants distinguished between receiving and giving feedback as part of formal processes and/or as part of the daily interactive flows. Informal comments or advice as part of interactive flows were noted as important for maintaining a positive ward climate and safe emotional environment. Informal “in the flow” feedback was mostly discussed by participants working at the frontline of daily care for patients:

**P4: I’ve got to be honest some days (……) some patients trigger other patients and then you’ve got three different incidents going on. Sometimes it’s impossible for the shift to run smoothly. However, the team I work with (….) we all work really well together and there’s definitely more informal feedback, than formal feedback, so if I manage to de-escalate a patient the nurse might come up to me and say ‘well done for that’ (….) the little comments that are made. Yeh, that happens quite a lot, we always try to say ‘you’ve done really well’, or ‘thank you for doing that’, ‘you’ve dealt with that really well’, little comments like that. I: What you’re saying is those little comments are quite important? P4: Oh yes, they bring your mood right up, because sometimes you feel like you’re not making a difference (….). Little comments, they make a massive difference.”**

The example below of an SSW providing feedback when they felt a staff/patient relationship was in danger of “‘crossing boundaries” suggests that knowing what is “going on” when you are in the flow is not always clear for those monitoring the flow of daily care. Being part of interactive flows often leaves little time to clarify the meaning of observations and make decisions:

**P9: (If a patient is) requesting the same person all the time we’ll explain that they need to work with every staff member and to build these therapeutic relationships (……) And, obviously sometimes if you’re with the same patient all the time like boundaries can be crossed and they may think it’s more of a friendship than a staff to patient relationship, (I: asks how the feedback is given to staff) I’d say that’s less formal. I wouldn’t wait for the monthly supervision to say, bring that up I’d probably do it, if I felt like it on the day, I would speak to that staff member then, I wouldn’t wait (…) relationships could worsen.”**

What gets noticed within the interactive flows acts as feedback influencing staff practice, and what gets lost is unknown. As one participant noted regarding the difference in how “scores” and “comments” in outcome reports are used:

**P5: That was definitely true for that outcome (quarterly report) stuff. Nothing gets done with those comments (….) the scores quantitative stuff, that gets put into an Excel sheet, gets presented and the comments are just lost in the ether.”**

#### Subtheme: learning how to practice from watching others

Some participants reported that they learnt how to practice—to interact with patients, particularly during emotionally tense situations, i.e., “incidents”, by watching how other staff managed, then “trying this out” in practice. In line with the broad definition of feedback, this acts as an addition to more formal feedback such as discussion in de-brief sessions to assist learning and desired practice change—for example:

**I: (Let’s) talk about how they learnt to do the job. P2: I’ve definitely done it by watching and observing, just absorbing every bit of information I can. Even when it comes to de-escalation and things like that, I obviously saw what worked in that moment, with that particular patient, might not work for another one. So it’s just learning all the things like that, how you’d speak to one patient is not necessarily how you’d speak to another. Maybe a bit more firm with one than with another as it’s what works for them. It’s literally about observation, taking it all in and then trying it yourself when you become a more permanent staff member and the person’s built a bit of a therapeutic working relationship with you and trusts you a bit more. There are aspects where you try and do it your own way as well, see what works for you, but definitely a lot of it is watching and learning.”**

Individual style and “tacit” intuitive skills interacted with objective learning of professional knowledge and skills in working practice. Feedback is sometimes aimed to changing or recognizing useful intuitive tacit interactive skills:

**P5: So after that feedback (…) I tried to be more open with how I was feeling, because they (supervisor) said they would rather know I was indecisive because of XYZ rather than assuming, so I guess I try to be a bit more open with whatever it is. But with the positive feedback as well, I’ve tried to use that to maintain whatever is obviously working for me. So things like my authentic interactions with people (….).**

#### Subtheme: shared experiences influence how feedback is perceived

Some participants felt that feedback from colleagues with shared work experiences had greater value than feedback based upon the providers’ position or qualification:

**P4: I think it definitely makes a difference if it comes from somebody who’s working alongside you. I think sometimes if management is ‘oh well done for that’, even though it feels good, I think sometimes it’s a little bit like ‘but he wasn’t there’, do you know what I mean? When it comes from somebody who was there, I think it can sometimes mean a bit more. Like I said, I would much prefer to do my supervisions with a senior than my unit manager, because they get it.”**

#### Subtheme: feedback is emotional as well as behavioral

Feedback literature focuses upon its use to achieve specific staff practices, i.e., behaviors. Yet for many participants, especially for frontline staff, feedback occurred during emotionally charged situations and was focused upon achieving emotional and physical safety for staff and patients. Informal immediate feedback was important for individuals and for its general contribution to the ward environment or “ward climate”.

**P7: I think I always find it differs and it can become complex and I think sometimes, my biggest hurdle with feedback sometimes is obviously that emotion behind it (……) because it’s theirs and it’s how they feel.**

**P8: Because I think in these sessions, you often become a kind of separate space for the patients where they can talk to you about the difficulties they’re having and that often does relate to staff and they’ll say, oh, you know, ‘they didn’t give me any talk time, they weren’t supporting me.’ So having to feed that back to the ward staff, knowing the stress that they’re under and the long days, you know how hard it is, broaching that is really, really tough…**

### Theme 3: feedback experiences differ along professional lines

Marked differences in feedbacks were seen between frontline clinical psychology staff and nursing support staff.

Nursing support staff often experienced feedback as informal and unplanned within interactive flows, focused upon maintaining emotional/physical safety, managing challenging behaviors, and maintaining hospital routines. This feedback appeared less focused upon planned development of practice skills than managing immediate challenges, e.g., self-injury and aggression. In this example, a staff nurse talked about directing SW to prevent challenging behaviors:

**P6: Even support workers that have been working for a while. You get situations where you have to tell them, for instance when they get told you shouldn’t be here by a patient. “Listen, it’s not worth it, you have to disengage from the situation, he’s not in a frame of mind that you can discuss anything with him.”**

“De-escalation” and behavioral management for patients also included more planned feedback as in debriefing meetings for practice development and emotional support:

**P2: (debriefs …) are all a learning experience. If you can get to the bottom of the reasoning or rationale behind how that patient managed to tie a ligature or how they managed to block their airways, then you know how to risk assess a bit better I suppose. Nobody ever wants to see it but again I guess that’s how you learn.**

For psychology staff (AP/PPW) working directly with patients, feedback was usually planned, formal, and monthly focused upon providing clinical programs, e.g., fire setting and anxiety reduction and completing written clinical assessments and medico-legal reports, e.g., HS20 and Outcomes reports. From an AP recently, a PPW:

**P8: Um, I didn’t even realize it was part of my job description as a PPW, that the ward manager was supposed to do my supervisions; whereas that didn’t happen. But my psychology supervisions in providing feedback have always been timely, even as a PPW I would have them monthly and that’s continued now in my assistant psychologist role. But they’ve been, from a psychological perspective, you’re getting feedback from someone who’s got a much more, kind of higher expertise than you have. So, having them formulate patient experiences and difficulties, um from their perspective and their experiences, is so helpful in how I move forward with the patients as well. So, I think, if I didn’t have those clinical supervisions, then I’ve obviously got to have them, as I not qualified, but the impact on the work that I would do would be huge and they’ve been really valuable, yea.**

**P1: For example, I have sessions with one patient every Tuesday and she’s a learning disability and autistic patient, so the way we work with her is a bit different. I’m enjoying that as I’m getting to learn from E (clinical psychologist) how I would change the way I work with patients. I do really like working with this patient. Then everything I do in the session, I feed back to E (…) she’s also given me a structure where she’s said, when you’re on the ward can you observe this, so we know how to structure the next session? Can you observe if she’s using any of these skills?**

Other feedback intended to change practice is more planned and procedural for example, monthly positive monitoring and SSP reports:

**P1: I think that’s why the SSPs are really important, because one to one we do positive monitoring forms, which in small is feedback forms, so me or A would do them with the patient, one to one, and they give us their feedback on what’s working. It’s split into three (what’s working well, what needs to change, what doesn’t work) and we have 2 columns for each (staff feedback and patient feedback). So they’re really important. So then I would go write up (….) Me and A would write it into the monthly SSP and then staff are required to read that and sign it, to say that it is acknowledged.”**

### Theme 4: feedback and individuality

A few participants referred to feedback as providing reassurance regarding their role performance:

**P1: …but I kind of used it (supervision) as an hour to ask how I was doing and if I was actually doing the role correctly, so then I could learn. (….) Yeah, you don’t know until you ask, it’s all good me saying I’m fine, I’m doing this, I’m doing this, but I’m not going to know if I don’t ask.**

**P10: When I first qualified, I was obviously “impostor syndrome” and I was “What am I doing?” “rabbit in the headlights” can’t do, don’t know what I’m doing. My unit leader was “on me,” “you can do this,” “you can do that,” (….) I think it’s that belief. If somebody has that belief in you then you maybe start thinking “well, hang on a minute, maybe I can do this, maybe I can do that.”**

Feedback always interacted with individual personal factors, e.g.:

**P10: I was always very conscious of what kind of manager I wanted to be and what kind of manager I’m confident in being. If somebody said to me though “you’ve got to be firm, you got to do this, you’ve got to,” I wouldn’t feel comfortable doing that. Therefore, I wouldn’t feel confident doing that: therefore, I wouldn’t be doing my job properly.**

**P5: So after that feedback I tried to be less, I tried to be more open with how I was feeling, (….) so I guess I try to be a bit more open with whatever it is. But with the positive feedback as well, I’ve tried to use that to maintain whatever is obviously working for me. So things like my authentic interactions with people.**

## Discussion

A total of 11 staff within an acute MH hospital were interviewed in May 2024, and the audio-taped material was transcribed and subjected to thematic analysis, resulting in four main themes:

- The complexity of feedback environment which was positive and open;
- Feedback ranged from formal and procedural to informal feedback responsive to interactive flows;
- Feedback differed for frontline psychology and nursing practice support. Psychology feedback was focused upon delivering and monitoring clinical programs and provided within reports and regular, structured meetings. Nursing feedback was focused within immediate interactive flows during managing/preventing challenging behaviors and maintaining physical/emotional safety;
- Specific individual factors influenced experiences of feedback.

This study has limitations. It gathered data in one acute MH hospital, and the results do not necessarily generalize to other such services. The study was pragmatic and exploratory and changed its primary focus from organizational-level feedbacks to an exploration of the overall feedback environment. Collecting qualitative data within a busy acute MH hospital presented difficulties for enabling frontline staff time to attend interviews. This entailed a senior manager locating and facilitating voluntary participation as the study was being conducted despite prior dissemination of information and invitation to participate. This meant that some groups were not sampled, e.g., ward managers and support workers, i.e., pragmatic rather than theoretical sampling. Although data saturation (26) was reached for the professional groups interviewed, other data may have arisen from missing groups of staff, potentially leading to different thematic structures or additional themes. Information regarding the hospital or organizational policies, e.g., training program and general environment, were not collected apart from participants. Grounding data within frontline staff and managers’ experiences limit the understanding of potential linkages between some aspects of general policy and staff experience except in relation to selected targeted overall organizational-level feedback structures.

Interventions (such as recommend a colleague) may not have intended impact if they are not valued by the frontline staff. Lack of shared experiences between frontline staff and senior managers may be one factor reducing the impact of such feedback. However, they may contribute to an overall culture and positive organizational working environment. The evidence for impact of organization-level interventions for a variety of goals is mixed, e.g., improving “ward climate” (27) with implementation processes a noted problem for hospital-based interventions (28). The systematic review of Brown et al. (29) noted a lack of consistent outcomes and theory of “clinical performance feedback” and suggested a new complex theory to focus such feedback, while Deveau et al. (30) showed that senior leaders felt it was personal interactions with frontline staff, observations, and individual feedback that were more effective than more formal communications. This is clearly a useful area for further study.

Secondly, this study showed a clear distinction between feedback processes within two different working contexts for psychology versus nursing supports. Frontline nursing supports often related feedback as informal and responsive to events within relatively uncontrolled interactive flows of patient/staff groups. Frontline psychology support related feedback as planned, structured interactions within relatively controlled contexts. Daniel Kahneman’s (31) description of thinking is as two systems: system 1 and system 2, i.e., “fast and slow” thinking provides a concept for understanding of feedback in nursing contexts as focused within fast (system 1) thinking and in psychology contexts within slow (system 2) thinking. Kahneman (31) summarizes the two modes of thinking (referred to here as intuitive/unconscious thinking and rational/conscious thinking):

System 1 continuously generates suggestions for system 2: impressions, intuitions, intentions, and feelings. If endorsed by system 2, impressions and intuitions turn into beliefs, and impulses turn into actions” (Kahneman p 24). “…. In summary, most of what you think and do originate in your system 1, but system 2 takes over when things get difficult and it normally has the last word.” (Kahneman p 25).

Nursing support staff discussed feedback in rapidly changing situations, responding very quickly to patient behavior which presented risks to physical and emotional safety in intuitive thinking mode. Such rapidly changing contexts hinder the ability to bring into play slow conscious rational thinking as applied by frontline psychology staff.

For both frontline nursing support and psychology staff, conscious rational thinking may struggle to maintain primacy and control (having the last word) of their actions in non-crisis interactions, when relaxed comfortable face-to-face interaction is usually maintained intuitively—for example, when AP (P5, see below) says “I forget it when I come face to face with a human” and when SSW (P9, see above) discusses their thinking upon observing a SW “getting too close to a patient”. These examples show that even when conscious rational thinking is appropriate and possible, intuitive thinking is always working, providing “impressions, intuitions, intentions, and feelings” to guide interactions.

These results suggest that psychologists were more accustomed to providing/receiving feedback using slow conscious thinking and nursing supports using intuitive thinking. This appears to match the daily demands of their roles. However, factors other than daily context, not specifically explored in this study, are also likely to play a role in these observed differences, such as the higher educational and occupational status of psychologists. Different professional standards, guidance, norms, and work practices may also lead to different expectations, which reinforce rational conscious thinking or relational intuitive thinking. Nursing norms and practices may reinforce an immediate “caring” focus for interactions and psychology norms and practices, a more distant role as “therapist”.

Jackson and Stevenson (32) proposed mental health nurses relationships with patients, as being on a spectrum from professional, through pseudo professional to ordinary, subsequently mapped to Kahneman’s two systems of thinking (1) (see **Table 3**). This appears equally relevant for psychologists and other therapists.

**Table 3:** MHN engagement mapped to two systems of thinking adapted from (1).

Three subcategories – the three me’s (and their dimensions)		
Ordinary intuitive engagement	Ordinary/professional engagement	Professional engagement
Ordinary communication: lay language through informal concepts	Ordinary Communication: mingling professional concepts with everyday language	Communication: structured via professional concepts and jargon
Two systems of thinking and MHN engagement		
Engagement guided by intuitive thinking with lazy conscious oversight, feels effortless and comfortable. Including banter, humour, self-disclosure and informative responses to questions. System 1 has no means of alerting system 2 when it gets into trouble	System 2 ‘waking up’ to more effort required in guiding the engagement, may become noticeable to communication partner, as hesitancy. System 2 may not recognise its conscious, planned approach is required in any particular situation	Engagement is under system 2 effortful control, is consciously planned, and control is usually obvious to communication partners. Therapeutic engagement is structured and setting becomes contained within professional standards, training and supervision

Primacy is given to “professional me” and conscious rational thinking in professional and practice development, e.g., therapeutic relationships and incident management. Ordinary me and intuition are perceived as having a problematic or little role. However. Kahneman’s formulation of skilled intuition (i.e., repeated immediate unequivocal feedback in a regular practice environment, p. 414) suggests that the importance given by frontline staff to immediate feedback delivered within interactive flows is not misplaced. Kahneman (31) also provides a theoretical basis for largely practice-based interventions: according “situational” leadership during an “incident” (33) and training/modeling good staff practice with aggressive patients (34) upon staff with the best (largely intuitive) working relationship rather than the most senior or expert present.

While it feels appropriate to use conscious rational conscious thinking to improve intuitive responses—can intuition also improve conscious thinking, e.g., within controlled, structured therapeutic interactions? Deveau (1) provided two comprehensive examples where intuition, as a deviation from standardized professional practice, played a very positive role within: a therapy session and a challenging patient care situation. This study provided examples where the participants became aware of intuitive thinking which they responded to, whether helpful or not:

**I: Do you find that (clinical supervision) guides you at all in a helpful way to be empathetic but yet follow the sort of (clinical) program you’ve got in mind? P5: Maybe somewhat. Quite often whatever I had in mind has gone out of the window when I’m face to face with somebody.**

System 1 is awake and working effortlessly from the moment we wake up with effortful system 2 thinking getting involved when intuition leads to problems or in a practiced manner.

Thirdly, **Table 4** shows a spectrum of professional development activities and proximity (time) to events and focus for feedback. This study supports other research suggesting that qualified mental health nurses have limited access to clinical supervision ((CS) individual guided self-reflection)) in comparison to other health professions, e.g., psychology (35, 36). Unqualified nursing supports have rarely reported access to CS. **Table 4** suggests the main opportunity for reflection on practice by mental health nurses that occurs after “failings” in care is examined in de-briefing sessions organized and managed by managers or professionals not involved in “incidents” (e.g. 37).

**Table 4:** Proximity and focus related to type of feedbacks experienced by nursing and psychology supports.

Psychology	Nursing supports	Nursing supports	Nursing supports
Clinical supervision. Structured, regular, monthly, and expected professional practice	Managerial/nursing supervision. Sporadic, less than monthly (often much less) than expected	Debriefing (as soon after) an incident as possible, might be days	Within interactive flows, especially managing incidents and preventing escalation
Focus. Subjective and objective reflection of supervisees experiences and feelings	Focus. Practice problems, “personal issues”, knowledge of recent policies, and working relationships	Focus. Reflection on management of incidents particularly those with serious harmful outcomes	Coaching/teachable moments. Focus upon immediate events and emotional and physical safety

It is likely in these significant reflection events for nursing supports that intuitive practice is viewed as having little or negative effects (see example P6 below).

Intuitive expertise in nursing supports is as unique as individual life and work experiences. Some will have good intuitive skills for their work, others not so good. Examples of helpful de-escalatory interactions with distressed patients were reported in this study. Intuitive expertise/skills are seen as important sources of good mental health nursing practice, as “tacit’ skills” (38, 39), and have been employed to develop nursing practice for reducing restraints use in acute services (34).

Do reflective experiences separated from interactive flows have a beneficial effect upon practice within the flow? As in the example, (P6) above described difficulties (while present and observing) in prompting a SW to “withdraw and distance themselves” from an escalating confrontation with a patient. This suggests that linking feedback delivered within reflection (CS or managerial supervision, debriefing) to feedback during interactive flows should be more “joined up”. Develop the links and certainty between various forms of proximally distant feedback to immediate events—for example, P6 (staff nurse) above reported being “encouraged” to undertake a management supervision of SW but wanted to wait to link this with observed (i.e., not subjectively reported) SW practice: P6: “I’ve been observing support workers. When I feel like I can intervene that’s when I’ll do one (supervision) …. My first one I want it to be something specific situation, something the support worker has to change.”

Practice coaching has become a distinct professional speciality similar to CS (e.g., 40) but can also be regarded as immediate guidance within interactive flows, especially when delivered during *teachable moments* (TM). TM are defined as “a synchronous event involving the unpredictable opportunity for teaching, when the client is open and ready to learn, and the delivery of key information takes place.” (41 p.7). TM are recognized through a practitioner’s “knowledge, intuition, and identification of the openness, readiness, and possibility for learning to occur” (41). Lawson and Flocke (42) suggest that TM are not always unpredictable, that practitioners can be “cued” through awareness of their “perceptions, cognitions, and motivations” (p5) to “create” a TM. This suggests that system 2 maybe cued to become active by the presence of certain intuitive factors and take over the interaction, creating a TM. Both Baker (41) and Lawson and Flocke (42) propose TM as opening the way for learning new (health) behaviors because of the immediacy of heightened emotional experience within which feedback is delivered. This reflects the immediacy of feedback delivered within interactive flows as part of emotional events shown by nursing supports in this study.

## Conclusions

This study showed that frontline staff in one acute MH unit (and likely to reflect many other such services) work in a complex feedback environment. Simple organization-level feedbacks showed limited impact upon frontline staff. However, these may have contributed to the overall positive hospital climate.

The study suggested feedback as complex encompassing a great deal of the communication behavior within a setting. Feedback within frontline nursing and psychology supports were focused within different contexts and employed different modalities. Nursing within immediate interactive flows while managing physical and emotional risks and providing daily care where feedback was responsive, informal, and verbal. Psychology feedback was regular, formal, and focused upon delivery of structured programs, verbal and written.

It is suggested that Daniel Kahneman’s two systems of thinking—rapid, unconscious, intuitive and slow/conscious and rational—is a useful approach to understanding these differences within nursing and psychology feedback contexts and experiences.

Further research is needed to explore the links between feedbacks that are delivered during practice and within formal clinical or management supervision. In particular, Lawson and Flocke’s (42) teachable moments might be used to help develop what Kahneman refers to as developing intuitive expertise through experiencing immediate feedback in a regular environment. The bringing together of formal (distal) and immediate (in the moment) feedback and development of these as part of a single system of feedback through which practice is developed could enhance the quality of support provided and develop the skills of practitioners.

## Data Availability

The raw data supporting the conclusions of this article will be made available by the authors, without undue reservation.

## References

[1] Deveau R. Everyday ordinariness, neglected but important for mental health nurses’ therapeutic relationships: An initial exploration for applying Daniel Kahneman’s two systems of thinking. Int J Ment Health Nurs. (2024) 33:369–77. doi: 10.1111/inm.13239, PMID: 37811594

[2] Rydon SE. The attitudes, knowledge and skills needed in mental health nurses: The perspective of users of mental health services*. Int J Ment Health Nurs. (2005) 14:78–87. doi: 10.1111/j.1440-0979.2005.00363.x, PMID: 15896254

[3] KUSHLICK A. Some ways of setting, monitoring and attaining objectives for services for disabled people. Br J Ment Subnormal. (1975) 21:84–102. doi: 10.1179/bjms.1975.017

[4] Mehner L Rothenbusch S Kauffeld S. How to maximize the impact of workplace training: a mixed-method analysis of social support, training transfer and knowledge sharing. Eur J Work Organization Psychol. (2025) 34:201–17. doi: 10.1080/1359432X.2024.2319082

[5] Li C Li L Wang Z. Knowledge, attitude and behaviour to evidence-based practice among psychiatric nurses: A cross-sectional survey. Int J Nurs Sci. (2022) 9:343–9. doi: 10.1016/j.ijnss.2022.06.016, PMID: 35891916 PMC9305010

[6] Jones E Felce D Lowe K Bowley C Pagler J Gallagher B. Evaluation of the dissemination of active support training in staffed community residences. Am J Ment Retard. (2001) 106:344–58. doi: 10.1352/0895-8017(2001)106<0344:EOTDOA>2.0.CO;2, PMID: 11414875

[7] Jones E Felce D Lowe K Bowley C Pagler J Strong G. Evaluation of the dissemination of active support training and training trainers. J Appl Res Intellect Disabil. (2001) 14:79–99. doi: 10.1046/j.1468-3148.2001.00064.x

[8] Gormley L Healy O O’Sullivan B O’Regan D Grey I Bracken M. The impact of behavioural skills training on the knowledge, skills and well-being of front line staff in the intellectual disability sector: A clustered randomised control trial. J Intellect Disabil Res. (2019) 63:1291–304. doi: 10.1111/jir.12630, PMID: 31106922

[9] Mansell J Beadle-Brown J. Active support: Enabling and empowering people with intellectual disabilities. London: Jessica Kingsley (2012).

[10] Mansell J Hughes H McGill P. Maintaining local residential placements. In Emerson E McGill P Mansell J (Eds.), Severe learning disabilities and challenging behaviours: Designing high‐quality services (pp. 261–281). Chapman and Hall, London.

[11] Mansell J Beadle-Brown J Ashman B Ockendon J. Person-centred active support: a multi-media training resource for staff to enable participation, inclusion and choice for people with learning disabilities. Brighton, UK: Pavilion (2004).

[12] Bould E Beadle-Brown J Bigby C Iacono T. The role of practice leadership in active support: Impact of practice leaders’ presence in supported accommodation services. Int J Dev Disabil. (2018) 64:75–80. doi: 10.1080/20473869.2016.1229524, PMID: 34141293 PMC8115458

[13] Cleary M Horsfall J O’Hara-Aarons M Hunt GE. Leadership, support and acknowledgement of registered nurses work in acute mental health units. Int J Ment Health Nurs. (2012) 21:445–52. doi: 10.1111/j.1447-0349.2011.00804.x, PMID: 22554252

[14] Stuber F Seifried-Dübon T Rieger MA Gündel H Ruhle S Zipfel S. The effectiveness of health-oriented leadership interventions for the improvement of mental health of employees in the health care sector: a systematic review. Int Arch Occup Environ Health. (2021) 94:203–20. doi: 10.1007/s00420-020-01583-w, PMID: 33011902 PMC7532985

[15] Pedersen MS Landheim A Møller M Lien L. Audit and feedback in mental healthcare: staff experiences. Int J Health Care Qual Assur. (2018) 31:822–33. doi: 10.1108/IJHCQA-08-2017-0142, PMID: 30354880 PMC6290895

[16] Hoben M Ginsburg LR Easterbrook A Norton PG Anderson RA Andersen EA. Comparing effects of two higher intensity feedback interventions with simple feedback on improving staff communication in nursing homes—the INFORM cluster-randomized controlled trial. Implement Sci. (2020) 15:1–16. doi: 10.1186/s13012-020-01038-3, PMID: 32912323 PMC7488270

[17] Nazir A Unroe K Tegeler M Khan B Azar J Boustani M. Systematic review of interdisciplinary interventions in nursing homes. J Am Med Directors Assoc. (2013) 14:471–8. doi: 10.1016/j.jamda.2013.02.005, PMID: 23566932

[18] Jones J Bion J Brown C Willars J Brookes O Tarrant C. Reflection in practice: How can patient experience feedback trigger staff reflection in hospital acute care settings? Health Expectations (2019) 23(2):396–404., PMID: 31858677 10.1111/hex.13010 PMC7104653

[19] Sheard L Marsh C O’Hara J Armitage G Wright J Lawton R. The patient feedback response framework–understanding why UK hospital staff find it difficult to make improvements based on patient feedback: a qualitative study. Soc Sci Med. (2017) 178:19–27. doi: 10.1016/j.socscimed.2017.02.005, PMID: 28189820 PMC5360173

[20] Sheard L Peacock R Marsh C Lawton R. What’s the problem with patient experience feedback? A macro and micro understanding, based on findings from a three-site UK qualitative study. Health Expect. (2019) 22:46–53. doi: 10.1111/hex.12829, PMID: 30244499 PMC6351417

[21] Bacotti JK Grauerholz-Fisher E Morris SL Vollmer TR. Identifying the relation between feedback preferences and performance. J Appl Behav Anal. (2021) 54:668–83. doi: 10.1002/jaba.804, PMID: 33440028

[22] Matey N Sleiman A Nastasi J Richard E Gravina N. Varying reactions to feedback and their effects on observer accuracy and feedback omission. J Appl Behav Anal. (2021) 54:1188–98. doi: 10.1002/jaba.840, PMID: 33856045

[23] Ramanadhan S Revette AC Lee RM Aveling EL. Pragmatic approaches to analyzing qualitative data for implementation science: an introduction. Implement Sci Commun. (2021) 2:p.70. doi: 10.1186/s43058-021-00174-1, PMID: 34187595 PMC8243847

[24] Braun V Clarke V. Using thematic analysis in psychology. Qual Res Psychol. (2006) 3:77–101. doi: 10.1191/1478088706qp063oa, PMID: 32100154

[25] Braun V Clarke V. To saturate or not to saturate? Questioning data saturation as a useful concept for thematic analysis and sample-size rationales. Qual Res Sport Exercise Health. (2021) 13:201–16. doi: 10.1080/2159676X.2019.1704846

[26] Braun V Clarke V. One size fits all? What counts as quality practice in (reflexive) thematic analysis? Qual Res Psychol. (2021) 18:328–52. doi: 10.1080/14780887.2020.1769238

[27] Dickens GL Johnson A Steel K Everett B Tonkin M. Interventions to improve social climate in acute mental health inpatient settings: Systematic review of content and outcomes. SAGE Open Nurs. (2022) 8:23779608221124291. doi: 10.1177/23779608221124291, PMID: 36533258 PMC9749049

[28] Geerligs L Rankin NM Shepherd HL Butow P. Hospital-based interventions: a systematic review of staff-reported barriers and facilitators to implementation processes. Implement Sci. (2018) 13:1–17. doi: 10.1186/s13012-018-0726-9, PMID: 29475440 PMC5824580

[29] Brown B Gude WT Blakeman T van der Veer SN Ivers N Francis JJ. Clinical Performance Feedback Intervention Theory (CP-FIT): a new theory for designing, implementing, and evaluating feedback in health care based on a systematic review and meta-synthesis of qualitative research. Implement Sci. (2019) 14:1–25. doi: 10.1186/s13012-019-0883-5, PMID: 31027495 PMC6486695

[30] Deveau R Gore N McGill P. Senior manager decision-making and interactions with frontline staff in intellectual disability organisations: A Delphi study. Health Soc Care Commun. (2020) 28:81–90. doi: 10.1111/hsc.12842, PMID: 31482622

[31] Kahneman D. Thinking, fast and slow. New York: macmillan (2011).

[32] Jackson S Stevenson C. What do people need psychiatric and mental health nurses for? J Adv Nurs. (2000) 31:378–88. doi: 10.1046/j.1365-2648.2000.01288.x, PMID: 10672096

[33] Ravoux P Baker P Brown H. Thinking on your feet: understanding the immediate responses of staff to adults who challenge intellectual disability services. J Appl Res Intellect Disabil. (2012) 25:189–202. doi: 10.1111/j.1468-3148.2011.00653.x, PMID: 22489031

[34] Bowers L James K Quirk A Simpson A Stewart D Hodsoll J. Reducing conflict and containment rates on acute psychiatric wards: The Safewards cluster randomised controlled trial. Int J Nurs Stud. (2015) 52:1412–22. doi: 10.1016/j.ijnurstu.2015.05.001, PMID: 26166187 PMC4518134

[35] McDonough JH Rhodes K Procter N. Impact of clinical supervision on the mental health nursing workforce: a scoping review protocol. BMJ Open. (2024) 14:p.e078765. doi: 10.1136/bmjopen-2023-078765, PMID: 38531589 PMC10966816

[36] Howard V Eddy-Imishue GEK. Factors influencing adequate and effective clinical supervision for inpatient mental health nurses’ personal and professional development: An integrative review. J Psychiatr Ment Health Nurs. (2020) 27:640–56. doi: 10.1111/jpm.12604, PMID: 31981445

[37] Mangaoil RA Cleverley K Peter E. Immediate staff debriefing following seclusion or restraint use in inpatient mental health settings: a scoping review. Clin Nurs Res. (2020) 29:479–95. doi: 10.1177/1054773818791085, PMID: 30051734

[38] Gobbi M. ‘The hidden curriculum’: Learning the tacit and embodied nature of nursing practice. In: Work-based learning in clinical settings. Florida, USA: CRC Press (2021). p. 103–24.

[39] Welsh I Lyons CM. Evidence-based care and the case for intuition and tacit knowledge in clinical assessment and decision making in mental health nursing practice: an empirical contribution to the debate. J Psychiatr Ment Health Nurs. (2001) 8:299–305. doi: 10.1046/j.1365-2850.2001.00386.x, PMID: 11882142

[40] Forge J. Ebook: Coaching in Mental Health Service Settings and Beyond: Practical Applications. Maidenhead UK: McGraw-Hill Education (UK (2022).

[41] Baker PW. An Educational Intervention to Improve Nurses’ Ability to Identify Behavioral Indicators of a Teachable Moment. ProQuest LLC, Ann Arbor, Michigan: Widener University (2022).

[42] Lawson PJ Flocke SA. Teachable moments for health behavior change: a concept analysis. Patient Educ Couns. (2009) 76:25–30. doi: 10.1016/j.pec.2008.11.002, PMID: 19110395 PMC2733160

